# Impaired Exercise‐Induced Mitophagy in Aged Muscle Coincides With Altered mitoAMPKα in Male Mice

**DOI:** 10.1111/acel.70827

**Published:** 2026-11-09

**Authors:** Niloufar Amiri, Anna S. Nichenko, Alex Churchill, Orion S. Willoughby, Torin D. Pettit, Charles P. Najt, Ricard A. Rodriguez‐Mias, Judit Villen, Joshua C. Drake

**Affiliations:** ^1^ Department of Human, Nutrition, Food and Exercise Virginia Polytechnic Institute and State University Blacksburg Virginia USA; ^2^ Institute for Critical Technology and Applied Science Virginia Polytechnic Institute and State University Blacksburg Virginia USA; ^3^ Metabolic Phenotyping Core Virginia Polytechnic Institute and State University Blacksburg Virginia USA; ^4^ Department of Genome Sciences University of Washington Seattle Washington USA

## Abstract

We demonstrate that the capacity for acute exercise to induce mitophagy in skeletal muscle is impaired in old male mice and corresponds to a loss of mitoAMPKα. We present evidence of age‐dependent, exercise‐induced phosphorylation of AMPK at S491, which may predispose mitoAMPKα to degradation by the E3‐ubiquitin ligase Trim72. Our findings highlight the need to understand how aging alters spatial responses to energetic stressors, like exercise.

## Graphical abstract

We demonstrate that the capacity for acute exercise to induce mitophagy in skeletal muscle is impaired in old male mice and corresponds to alterations in mitoAMPKα. Our findings highlight the need to understand spatial responses to energetic stressors, like exercise, with age for the maintenance of skeletal muscle health.

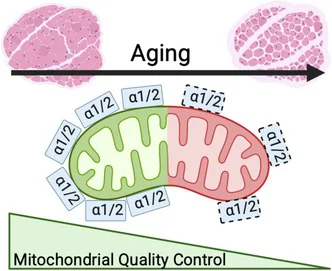

Aging is associated with a progressive decline in skeletal muscle function that contributes to frailty, reduced mobility, and metabolic dysfunction (Wilkinson et al. 2018). A hallmark of the functional decline of skeletal muscle is impaired mitochondrial energy production, which is observed across multiple tissues with age but is strongly linked to impaired muscle function and loss of muscle mass with age, known as sarcopenia (Janssen et al. 2025; Ferri et al. 2020). In skeletal muscle, maintenance of mitochondrial energy production is essential due to skeletal muscle's high energetic demand during prolonged contraction and central role in whole‐body metabolism (Craige et al. 2024; Nichenko et al. 2022). Mitochondria are preserved through synergistic processes, including biogenesis, fission/fusion dynamics, and recognition and removal of damaged/dysfunctional mitochondria (i.e., mitophagy), collectively referred to as mitochondrial quality control (Liu et al. 2023; Xie et al. 2023). Lifelong, regular exercise maintains skeletal muscle mass with age, in part, through upregulation of mitochondrial quality control processes (Laurin et al. 2019). Acutely, exercise upregulates each facet of mitochondrial quality control (Drake et al. 2016). With aging, skeletal muscle mitochondrial biogenesis does not decline, nor does the capacity for biogenesis to be stimulated by exercise (Miller et al. 2012; Robinson et al. 2017). However, skeletal muscle mitochondrial protein damage accumulates with age, consistent with an age‐related decline in macroautophagy/mitophagy in skeletal muscle (Drake and Yan 2017). It remains unclear whether the capacity for exercise to upregulate mitophagy in skeletal muscle declines with age.

We have previously demonstrated a 90‐min, progressive intensity treadmill running protocol that induces skeletal muscle mitophagy in mice (Laker et al. 2017). As exercise capacity declines with age in mice as it does in humans (Pajski et al. 2025; Westerstahl et al. 2025), exercise intensity must be normalized between young and old animals to evaluate age‐related differences in exercise response. We have shown mouse VO_2_ plateaus during progressive intensity sub‐maximal running that has been used to induce mitophagy (Laker et al. 2017) rather than display intensity‐dependent increases in VO_2_ (Amiri et al. 2025). Therefore, to test for age‐dependent changes in exercise‐induced mitophagy in skeletal muscle, we adjusted treadmill running speed based on the percent difference in body weight between old (24‐month‐old) and young (3‐month‐old) mice (Figure 1A). Adjusting running speed, while maintaining total running time and duration of speed intervals, which all mice completed, ensured both young and old mice performed equivalent work (*J*) (Bernier et al. 2022) (Figure 1B). Although old mice displayed a trend for lower lactate compared to young, there was no difference in the delta for pre/post blood lactate with exercise (Figure 1C,D). Therefore, work normalization based on difference in body mass, while not accounting for the myriad of differences that may influence exercise response with age (Janssens et al. 2026), may allow for comparisons of age‐dependent responses to acute exercise in mouse models.

**FIGURE 1:**
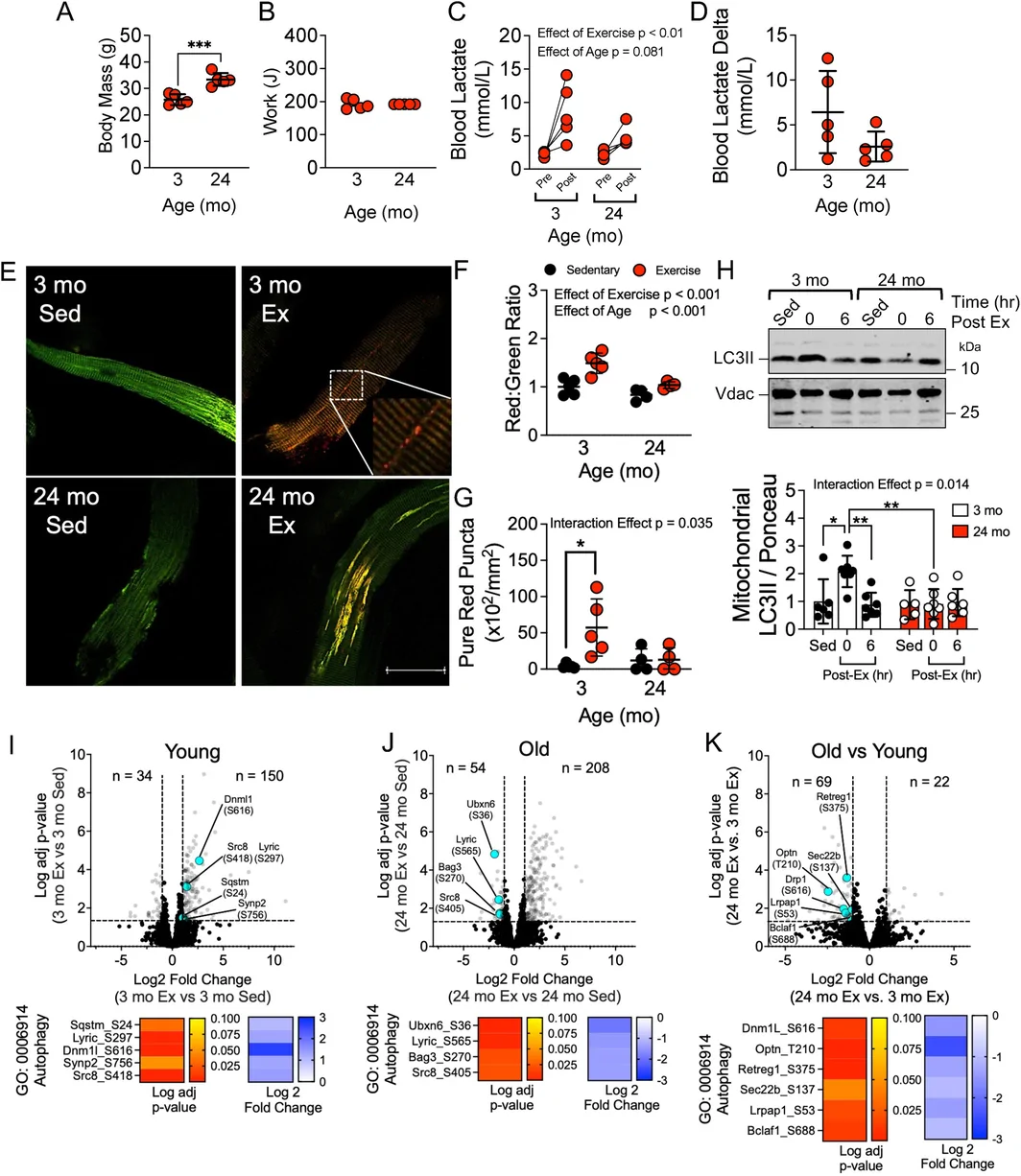
Exercise‐induced mitophagy in skeletal muscle is impaired with age. (A) Body mass between 3‐ and 24‐month‐old cohort. (B) Work performed during 90 min acute treadmill running when treadmill speed is normalized by percent difference in body mass. (C) Blood lactate Pre/post work‐normalized treadmill running. (D) Blood lactate delta pre/post work‐normalized treadmill running. 3‐month = 5, 24‐month = 5. (E) Representative MitoTimer images. (F) MitoTimer Red:Green Ratio. (G) MitoTimer pure red puncta (mitophagy events). 3‐month = 5, 24‐month = 4. (H) LC3II immunoblot in isolated mitochondria from GA muscle. *n* = 5–7 per timepoint or age. (I–K) Volcano plots comparing differences in plantaris phosphoproteome between groups. The number (*n*) of significant phospho‐events are indicated in gray. *n* = 5 per age and group. Data presented as mean ± SD. Main or interaction effects from Two‐way ANOVA are reported in each panel where applicable. *p < 0.05; **p < 0.01.

We directly assessed exercise‐induced mitophagy ex vivo via MitoTimer imaging of transfected flexor digitorum brevis (FDB) muscle (Laker et al. 2017, 2014; Drake et al. 2021) in young and old mice following work‐normalized acute exercise. FDB muscles were collected 6 h post‐exercise, corresponding to the peak in acute exercise‐induced mitophagy (Laker et al. 2017; Drake et al. 2021; Guan et al. 2024), and imaged via confocal microscopy for MitoTimer Red:Green ratio (oxidation dependent color change) and pure red puncta (mitophagy events) (Laker et al. 2017, 2014; Guan et al. 2024) (Figure 1E–G). Exercise induced a shift in the MitoTimer Red:Green ratio, as previously observed (Laker et al. 2017; Drake et al. 2021; Guan et al. 2024), though this was less pronounced in old mice (Figure 1E,F). This may allude to a difference in intensity despite equivalent work performed, or age‐related differences in dehydrogenization of the MitoTimer reporter in the matrix that is required for the fluorescent shift to red (Laker et al. 2014). Acute exercise increased mitophagy in young mice, as evidenced by an increase in pure red MitoTimer puncta (Figure 1E,G), as previously observed (Laker et al. 2017; Drake et al. 2021; Guan et al. 2024). However, exercise‐induced mitophagy was blunted in old mice, as indicated by a lack of exercise‐induced increase in pure red puncta (Figure 1E–G).

To validate our interpretation of impaired exercise‐induced mitophagy in old mice via MitoTimer imaging, mitochondrial‐enriched fractions were isolated from gastrocnemius (GA) muscle at 0 and 6 h post‐exercise (Laker et al. 2017; Drake et al. 2021). LC3II expression, a marker of mature autophagosome formation that localizes to mitochondria for mitophagy (Laker et al. 2017), was increased at 0 h after acute exercise in young mice (Figure 1H). However, old mice had no exercise‐induced increase in LC3II in mitochondrial‐enriched fractions post exercise (Figure 1H). Moreover, phosphoproteomic analysis of plantaris muscle immediately following acute exercise (0 h) revealed significantly increased phosphorylation of autophagy‐related proteins in young mice, according to gene ontology classification (Figure 1I). In contrast, phosphorylation of autophagy‐related proteins according to gene ontology classification in skeletal muscle of old mice was downregulated following acute exercise (Figure 1J). Impaired mitophagy/autophagy was further supported by comparison of old and young phosphoproteomes immediately post‐acute exercise, with autophagy‐ and mitochondrial fission‐related phosphorylation events downregulated in old mice compared to young in response to acute exercise (Figure 1K).

SQSTM1/p62 is a ubiquitin‐binding selective‐autophagy adaptor implicated in cargo recognition and recruitment of autophagic membranes to damaged mitochondria (Sun et al. 2026) and its phosphorylation was identified to be increased in young mice following acute exercise (Figure 1I). Immunoblotting of whole gastrocnemius lysates showed exercise increased p62 expression, but old mice had less expression of p62 compared to young (Figure S2A,B). As p62 has been shown to coordinate clustering of damaged/dysfunctional mitochondria during Pink1/Parkin‐mediated mitophagy (Sun et al. 2026), the age‐related decline in p62 expression supports the notion of impaired mitophagy capacity in old mice, although the role of Pink1/Parkin in exercise‐induced mitophagy is less clear (Drake et al. 2019; Seabright et al. 2020). Phosphorylation of Drp1 at Ser616 facilitates mitochondrial fission (Hanada et al. 2020; Kubli and Gustafsson 2012) resulting in translocation of Drp1 to the outer mitochondrial membrane in response to energetic stress (Toyama et al. 2016) and was identified to be upregulated with exercise in plantaris of young mice but downregulated in old mice following exercise relative to young counterparts (Figure 1I,K). In contrast to phosphoproteomics of plantaris, p‐Drp1(S616) in whole GA lysates increased following exercise in both young and old GA muscle with no difference between age groups (Figure S2A,C). However, expression of Drp1 in enriched mitochondrial lysates from GA muscle was significantly less in old mice (Figure S3D,E). As Drp1 must translocate from the cytosol to the outer mitochondrial membrane to drive fission (Zerihun et al. 2023), this finding suggests aging may negatively affect Drp1 recruitment to mitochondria and/or docking with mitochondrial outer membrane receptors which may negatively affect fission capacity in response to exercise. However, intravital imaging has shown increased mitochondrial fission following exercise in old mice despite lower expression of Drp1 and associated fission regulators (O'Reilly et al. 2025). In sum, we provide microscopic, protein expression, and phosphoproteomic evidence that the capacity for acute exercise to induce mitophagy in skeletal muscle is diminished in old mice and is associated with potentially impaired initiation of mitochondrial fission when exercise intensity is normalized to ensure equivalent work is performed between young and old mice.

To investigate possible mechanisms underlying impaired induction of mitophagy in skeletal muscle of old mice following acute exercise, we interrogated signaling responses to acute exercise in the plantaris phosphoproteome. We found 42 downregulated and 101 upregulated unique phosphorylation events in plantaris of old mice that were not significantly regulated in young mice (Figure 2A), suggesting the signaling response in skeletal muscle to work‐normalized acute exercise differs between young and old male mice. Consistent with altered molecular response to exercise in young and old humans (Janssens et al. 2026), altered skeletal muscle protein phosphorylation response to acute exercise may reflect the different physiological environments between 3‐ and 24‐month‐old mice. KEGG Pathway analysis of the 42 unique downregulated phosphorylation events in old mice revealed proteins related to cytoskeleton in muscle cells (Figure S3). KEGG Pathway analysis of the 101 unique upregulated phosphorylation events in old mice revealed proteins related to insulin resistance and longevity‐related signaling pathways, among others (Figure 2B). A common protein between identified KEGG pathways was AMPKα2, specifically phosphorylation at S491 (Figure 2C). Given that AMPKα2 S491 and the analogous AMPKα1 S485 inhibit AMPK activity (Wu et al. 2024; Jiang et al. 2021), these findings suggest dysregulation of energy sensing through AMPK signaling in response to acute exercise with aging.

**FIGURE 2:**
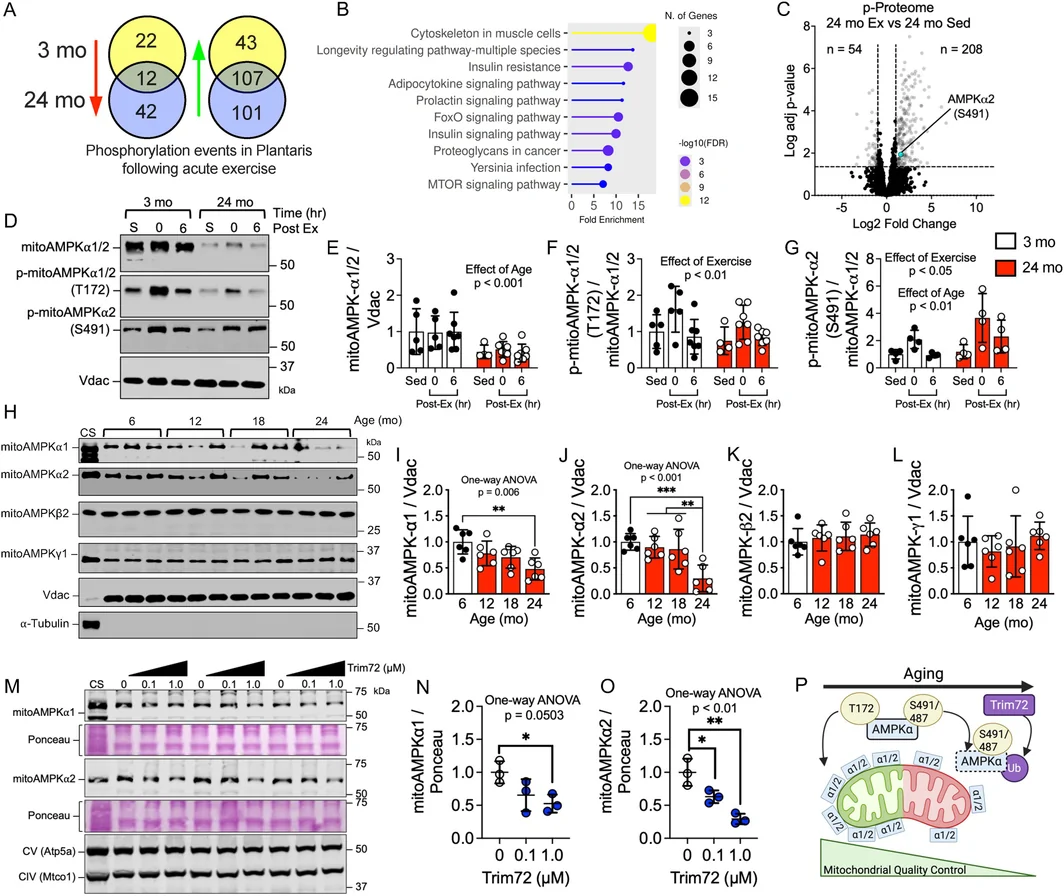
Loss of mitoAMPKα with age may be due to Trim72‐mediated degradation. (A) Venn diagram of significant phosphorylation events in plantaris muscle following work‐normalized acute exercise. Left: Downregulated with exercise. Right: Upregulated with exercise. (B) KEGG pathway analysis of significantly upregulated phosphorylation events unique to 24‐month‐old mice with acute exercise. (C) Volcano plot of 24‐month‐old phospho‐proteome highlighting AMPKα2 (S491). (D–G) Age‐related changes in mitoAMPK⍺ expression and T172 and S491 phosphorylation in GA muscle. *n* = 4–7 per age and timepoint. (H–L) Age‐related changes in mitoAMPK expression. *n* = 6 per age. (M–O) Skeletal muscle lysates from 3‐month‐old mice (*n* = 3) were combined, and aliquoted for three technical replicates per experiment and incubated with increasing concentrations of recombinant TRIM72, followed by mitochondrial isolations and immunoblot analysis of mitoAMPKα1 and mitoAMPKα2. ATP5A (Complex V) and MTCO1 (Complex IV) were assessed as mitochondrial protein controls. (P) Working model. Data presented as mean ± SD. Main or interaction effects from Two‐way ANOVA (E–G) or one‐way Anova (I–L, N, O) are reported in each panel where applicable. *p < 0.05; **p < 0.01; ***p < 0.001.

We have previously shown that a specialized pool of AMPK localized to the outer mitochondrial membrane (mitoAMPK) functions as a sensor of mitochondrial energetic stress and is required for exercise‐induced mitophagy (Drake et al. 2021). We therefore assessed mitoAMPKα expression and phosphorylation in mitochondrial enriched fractions isolated from GA muscle of young and old mice immediately (0 h) and 6 h post work‐normalized acute exercise. mitoAMPKα expression was significantly less in mitochondrial‐enriched fractions from GA of old mice compared to young (Figure 2D,E). Phosphorylation of the AMPKα subunit at Thr172 is required for full kinase activation and initiation of downstream pathways that promote mitochondrial quality control (Garcia and Shaw 2017). Furthermore, phosphorylation of mitoAMPKα1/2 at T172 is specifically responsive to mitochondrial energetic stress (Drake et al. 2021). We found mitoAMPKα1/2 T172 phosphorylation was increased immediately post‐exercise in both young and old mice (Figure 2D,F), though in context with less expression of mitoAMPKα in old mice (Figure 2D,E), this may indicate limited capacity for mitoAMPK‐mediated signaling. In contrast, phosphorylation of mitoAMPKα2 at Ser491, the inhibitory site identified in the phosphoproteomics (Figure 2C), was elevated in skeletal muscle immediately following exercise but more so in old mice (Figure 2D,G). Together these data suggest alterations to mitoAMPK with age that may limit its capacity to promote mitochondrial quality control processes, such as mitophagy (Drake et al. 2021).

As we found less expression of mitoAMPKα in skeletal muscle from old mice (Figure 2D,E) we next assessed whether aging differentially affects the abundance of individual mitoAMPK subunits. AMPK is a heterotrimeric complex composed of a catalytic α subunit and regulatory β and γ subunits, with skeletal muscle mitoAMPK composed of α1/2, β2, and γ1 subunits (Garcia and Shaw 2017). To determine whether aging selectively affects expression of specific mitoAMPK subunits, mitochondria were isolated from GA of 6‐, 12‐, 18‐, and 24‐month‐old mice. Expression of mitoAMPKα1/2 gradually declined with age, reaching significance by 24 months (Figure 2H–J) and correlated with declining GA mass (Figure S4A–C). However, expression of mitoAMPKβ2 and γ1 remained unchanged with age (Figure 2H,K,L). Interestingly, age‐related changes in AMPKα1/2 expression in whole GA lysates were not observed (Figure S4D–F), suggesting that the mitochondria‐localized mitoAMPKα1/2 are uniquely susceptible to age‐related degradation.

Phosphorylation of AMPKα2 at S491 has been shown to recruit the E3 ubiquitin ligase TRIM72, resulting in proteolytic degradation of AMPKα2 (Jiang et al. 2021). As phosphorylation of mitoAMPKα2 at S491 was elevated in old mice post exercise (Figure 2D,G), we tested if mitoAMPKα can be degraded by TRIM72. Skeletal muscle homogenates from 3‐month‐old mice were incubated ex vivo with increasing concentrations of recombinant TRIM72 (0, 0.1, 0.5, 1 or 2 mM) in the presence of TCEP, ATP, and ubiquitin, followed by mitochondrial isolation. Immunoblot analysis revealed a loss of both mitoAMPKα1 and α2 protein in the presence of TRIM72 (Figure 2M–O, Figure S5A–E). Whereas expression of inner mitochondrial membrane proteins, ATP5A (Complex V) and MTCO1 (Complex IV), remained unchanged across TRIM72 concentrations (Figure 2M), suggesting recombinant TRIM72‐mediated degradation of mitoAMPKα1/α2 was not due to generalized degradation of mitochondrial proteins.

Based on the present findings, we propose a working model in which age‐associated alterations in mitoAMPKα signaling and abundance may contribute to impaired exercise‐induced mitophagy in skeletal muscle (Figure 2P). Specifically, increased AMPKα2 S491 phosphorylation and the capacity for TRIM72‐mediated degradation may contribute to reduced mitoAMPKα abundance, thereby limiting downstream mitochondrial quality‐control signaling. Importantly, our findings were obtained only in male mice. Whether the age‐related loss of mitoAMPKα and the associated impairment in exercise‐induced mitophagy occur similarly in female skeletal muscle remains to be determined, and future studies including both sexes are needed. Further work is also needed to elucidate the localized pathways regulated by mitoAMPKα in skeletal muscle to discern the extent of impact for its loss with age as well as other, converging upstream signals that may predispose mitoAMPKα to degradation or dysregulation with age.

## 1 Methods

### 1.1 Animals

All animal procedures were conducted in accordance with protocols approved by the Virginia Tech Institutional Animal Care and Use Committee. All mice were housed in a temperature‐controlled environment (22°C) with a 12:12‐h light–dark cycle with ad libitum access to water and food (Envigo). Mice were not fasted prior to the acute exercise bout and had ad libitum access to food and water throughout the post‐exercise recovery period until tissue collection. Male C57BL/6J mice, aged 3 months (young) and 24 months (old), were obtained from NIA rodent colony at Charles River. Once received, mice were acclimated to the vivarium for 1 week before any manipulation.

### 1.2 Plasmid DNA and Transfection

The mitochondrial reporter MitoTimer was used to assess exercise‐induced mitophagy in vivo, as previous (Laker et al. 2017, 2014; Drake et al. 2021; Guan et al. 2024). *pMitoTimer* was delivered to the flexor digitorum brevis (FDB) muscle by somatic gene transfer. Mice were allowed to recover for 10 days prior to experimental interventions to ensure stable transgene expression and recovery from the procedure.

### 1.3 Acute Exercise

Afterwards, all mice were acclimated to treadmill running by running at 10 m/min on a 0° incline for 10 min for 3 consecutive days, as previous (Laker et al. 2017; Drake et al. 2021). Mice were then assigned to perform acute exercise on day 4 or remain sedentary. Young mice performed a 90‐min progressive treadmill running protocol to induce mitophagy that peaks 6 h into the recovery period post‐exercise (Laker et al. 2017; Drake et al. 2021; Guan et al. 2024). For all old mice randomly selected to perform acute exercise, body weight was recorded and running speed was adjusted based on the percent difference from average body mass of a cohort of young mice (Figure 1A). Body weight difference and normalized work calculation of all old mice that performed treadmill running relative to young cohort is found in Figure S6. Work performed during acute treadmill exercise was calculated as (Bernier et al. 2022):

$$\text{Work}\;\left(J\right)=\text{Weight}\;\left(\mathrm{kg}\right)\times \text{Distance}\;\left(m\right)\times \text{Time}\;\left(\min\right)\times 0.05\;\text{incline}$$

### 1.4 Confocal Microscopy

Animals transfected with MitoTimer were euthanized 6 h after completion of acute treadmill exercise to correspond to the peak in exercise‐induced mitophagy (Laker et al. 2017; Drake et al. 2021; Guan et al. 2024), FDB muscles were immediately fixed in 4% paraformaldehyde and whole‐mounted on gelatin‐coated slides using 50% glycerol in phosphate‐buffered saline. Confocal images were acquired at 25× magnification with 2× zoom (Leica SP8). All images were acquired using identical pre‐defined acquisition settings to avoid signal saturation and ensure consistent fluorescence intensity across samples. MitoTimer fluorescence was quantified using a custom‐designed Matlab‐based image analysis pipeline to determine red‐to‐green fluorescence ratios and the presence of mitophagy events via pure red puncta, as previous (Laker et al. 2017, 2014; Drake et al. 2021; Guan et al. 2024).

### 1.5 Mitochondrial Fraction Isolation

Mitochondrial‐enriched lysates were isolated from gastrocnemius muscles via differential centrifugation fractionation and prepared for western blotting, as previous (Laker et al. 2017; Drake et al. 2021).

### 1.6 Mass Spectrometry Sample Preparation

Both plantaris muscles were collected from 5 sedentary and exercised young and old mice and immediately frozen in LN2. Approximately 30 mg of frozen tissue (i.e., both plantaris from each mouse) was cryopulverized in liquid Nitrogen and powder resuspended in 600ul of denaturing lysis buffer (8 M urea, 75 mM Tris pH 8.2, 100 mM NaCl). 0.5 mm zirconia/silica beads were added to resuspended tissue and for four 1‐min bead cycles rounds carried out with 1 min on ice rest periods in between. Lysate was cleared by centrifugation at 21,000 *g* for 10 min at 4°C. Protein concentration was determined by bicinchoninic acid assay (BCA; Pierce, ThermoFisher Scientific). Proteins were reduced with 5 mM dithiothreitol for 30 min at 55°C, alkylated with 15 mM iodoacetamide for 30 min at room temperature in the dark, and quenched with an additional 5 mM dithiothreitol for 30 min at room temperature.

Lysates were processed with the R2‐P1 protocol (Leutert et al. 2019) implemented on a KingFisher Flex (ThermoFisher) magnetic particle processing robot with minor modifications. Briefly, 300 μg protein was combined with 900 μg carboxylated paramagnetic beads (1:1 mix of hydrophilic and hydrophobic Sera‐Mag SpeedBead Carboxylated‐Modified, GE Life Sciences) and ethanol was added to 80%. Proteins were washed four times in 80% ethanol before digestion in 50 mM triethylammonium bicarbonate pH 8.5 for 5 h at 37°C. Samples prepared to assess mistranslation frequency were digested with 5 ng/μL LysC (Wako Chemicals) and 5 ng/μL trypsin (Promega). Digests were acidified with formic acid (FA) and samples dried down.

For proteome analysis 20 μg digest was desalted over Empore MCX stage tips (Rappsilber et al. 2007). For phosphoproteome analysis, IMAC enrichment was carried out on 250 μg of dried digested peptides using the R2P2 protocol described by (Leutert et al. 2019). Briefly, peptides were resuspended in 80% acetonitrile (ACN) with 0.1% trifluoroacetic acid (TFA). Following clarification by centrifugation, phosphopeptide enrichment was performed on a KingFisher Flex with 75 μL 5% Fe^3+^‐NTA magnetic beads (PureCube Fe‐NTA MagBeads, Cube Biotech). After peptide incubation, beads were washed three times with 80% ACN, 0.1% TFA, and phosphopeptides eluted in 50% ACN, 2.5% NH4OH, acidified and dried down.

### 1.7 Mass Spectrometry Data Acquisition

Peptides were resuspended in 4% ACN, 3% FA and analyzed on an Orbitrap Exploris 480 Mass Spectrometer (ThermoFisher Scientific) equipped with an Easy1200 nanoLC system (ThermoFisher Scientific). Peptides were loaded onto a 100 μm ID × 3 cm precolumn packed with Reprosil C18 3 μm beads (Dr. Maisch GmbH) and separated by reverse‐phase chromatography on a 100 μm ID × 30 cm analytical column packed with Reprosil C18 1.9 μm beads (Dr. Maisch GmbH) housed in a column heater set at 50°C using two buffers: (A) 0.1% FA in water and (B) 80% ACN in 0.1% FA in water at 350 nL/min flow rate. Proteome digests were separated over a 73‐min 2 stage gradient consisting of 8% to 36% B in 65 min and 36% to 50% B in 8 min followed by wash and A reequilibration totalling 90 min. Phosphopeptides were separated over a 2 stage 73‐min gradient first 8% to 34% B in 66 min followed by 34% to 55% B in 7 min.

Data was collected using a data independent acquisition (DIA) method with staggered windows. In short, a full MS1 scan was recorded after every DIA cycle at 60,000 resolution with standard automatic gain control (AGC) target and automatic injection time (IT). Two distinct offset DIA cycles shifted by 12 m/z covered an effective range of 363 to 1095 m/z for proteome or 438 to 1170 m/z for phosphoproteome samples. Each DIA cycle contained 30 windows of 24 m/z size at 30,000 resolution, 30% or 27% normalized collision energy (NCE) higher‐energy collisional dissociation (HCD) for proteome and phosphoproteome samples, respectively. Finally, the default charge state was set to 3, and AGC target setting was 1000%.

### 1.8 Mass Spectrometry Data Analysis

MS raw files were converted to mzML and staggered DIA files demultiplexed using MSconvert v3.0.23305.fafbb32 (Adusumilli and Mallick 2017). Database search and spectral library search were performed using Fragpipe v22.0 (Yu et al. 2023) and Uniprot UP000000589 revision 2024‐12‐11 fasta database. In the searches, the maximum number of missed cleavages was set to 2, N‐terminal methionine excision was enabled, fixed modification on carbamidomethylated Cysteines was considered as well as variable modifications on M oxidation (15.9949 Da) and STY phosphorylation (79.9663 Da). DIA files were searched and quantified with DIA‐NN v2.1.0 (Demichev et al. 2020) using the spectral library created in FragPipe in “QuantUMS (high precision)” mode. For Phosphoproteome data, peptidoform scoring was enabled and 1% FDR and 90% site localization filters applied for STY phosphorylation. Further data analysis was performed using custom scripts in RStudio v2023.03.1 and R v4.3.0. Phosphosite intensities were log‐transformed prior to analysis. Differential abundance across experimental groups was assessed using linear models fit for each phosphosite with the *limma* package (Ritchie et al. 2015) in R, with empirical Bayes moderation of variance estimates used to calculate moderated *t*‐statistics and *p*‐values. *p*‐values were adjusted for multiple testing using the Benjamini‐Hochberg false discovery rate (FDR) method. Phosphosites were considered significantly regulated if they had an FDR‐adjusted *p*‐value < 0.05 and an absolute log2 fold change > 1.

### 1.9 Pathway Analysis of Phosphoproteomic Data

Identification of GO pathways was determined via ShinyGO v0.86.0 (https://bioinformatics.sdstate.edu/go/).

### 1.10 Western Blot

Western blotting was performed as described previously (Laker et al. 2017; Drake et al. 2021), using the following primary antibodies (1:1000 dilution): LC3I/II (CST #4108), phospho‐AMPK Thr172 (CST #2535), AMPKα1/2 (CST #2532), AMPKβ2 (Novus Biologicals #92286), AMPKγ1 (Abcam #ab32508), VDAC (CST #4866), α‐tubulin (Abcam #11304), and OXPHOS (Abcam #110413). Membranes were cut at appropriate molecular weight ranges following Ponceau‐S staining to allow probing of multiple targets on the same blot. Immunoreactive bands were visualized using the Odyssey infrared imaging system (LI‐COR). Protein abundance was quantified relative to a common internal protein standard loaded on each gel, which consisted of a whole‐tissue lysate mixture of liver, heart, and skeletal muscle.

### 1.11 Recombinant TRIM72 Expression and Purification

His‐SUMO‐TRIM72 was purchased from Addgene (#234225). Expression and purification was performed as previously described with modifications (Ma et al. 2023). In brief, tagged TRIM72 was transformed into *E. coli* BL21(DE3) Codon^+^ RIL (Agilent, Santa Clara, CA) and grown at 37°C in sterile LB media supplemented with 50 μg/mL kanamycin until OD_600_ ≈0.6. Once target OD_600_ was reached, cultures were cooled to 18°C and protein expression was induced by addition of 0.5 M IPTG. After 20 h, cells were harvested by centrifugation and re‐suspended in Buffer A (20 mM Tris–HCl, 0.2 M NaCl, 0.3 mM TCEP‐HCl, pH 8.0) with protease inhibitor cocktail (Halt EDTA‐free, 1:200) at 5 mL/mg. Re‐suspended cells were lysed using sonication. Lysates were clarified by centrifugation (4°C for 45 min at 12,000 *g*) and clarified lysate was loaded onto a 5 mL HisTrap HP column (Cytiva) equilibrated in Buffer A at 1 mL/min. The column was washed with 2 CVs of Buffer A, followed by a step gradient elution of 0 (10 CVs), 10 (10 CVs), 20 (5 CVs), 30 (5 CVs), 40 (10 CVs), and 100% (10 CVs) Buffer B (20 mM Tris–HCl, 0.2 M NaCl, 0.3 mM TCEP‐HCl, 250 mM imidazole, pH 8.0) at 5 mL/min. TRIM72 containing fractions were determined by UV280 signal and Western Blot and pooled. Pooled TRIM72 fractions were dialyzed against 20 diavolumes of Buffer A overnight at 4°C and diluted twofold in Buffer A. Eluted TRIM72 was further purified by 4 passes through an Amicon Ultra Centrifugal Filter, 100 kDa MWCO (Millipore), collecting the supernatant, to remove low molecular weight contaminants. Final purity was assessed by SDS‐PAGE and concentration determined by BCA assay.

### 1.12 Ex Vivo TRIM72 Incubation Assay

To assess the effect of TRIM72 on mitochondrial AMPK stability, skeletal muscles including quadriceps, gastrocnemius, soleus, and plantaris were processed for ex vivo incubation assays. Muscles from three mice, 3‐months‐of‐age, were homogenized in ice‐cold Tris–HCl buffer (pH 8.0) containing NaCl using an electrical homogenizer. Homogenates were centrifuged at 800 *g* for 10 min at 4°C to remove insoluble debris, and the resulting supernatants were collected as cleared lysates and pooled together. Cleared lysates were supplemented with ubiquitination reaction components, including ubiquitin (10 μM), ATP (5 mM), MgCl_2_ (5 mM), and TCEP (1 mM), and mixed thoroughly. Lysates were then equally divided into aliquots of 500 μL per tube, equating to technical triplicates for each intended concentration of recombinant TRIM72. Recombinant TRIM72 was added to lysates at final concentrations of 0 (control), 0.5, 1, or 2 (μM). Samples were incubated at 37°C for 1 h to allow TRIM72‐dependent ubiquitination reactions to occur. Following incubation, mitochondria were isolated from each sample by differential centrifugation as described above. Mitochondrial‐enriched fractions were subsequently analyzed by immunoblotting to assess changes in mitochondrial AMPKα protein abundance.

### 1.13 Statistics

Data are presented as mean ± SD and were analyzed and plotted via GraphPad Prism 9.5.1. Data were analyzed via Student's *t*‐test when one variable was present or two‐way ANOVA when two variables were present. Post hoc analyses were only performed when a significant interaction between a categorical and a quantitative variable was found, which are indicated in the figures where applicable. Statistical significance was established a priori as *p* < 0.05.

## Author Contributions

Study design: N.A., C.P.N., J.V., and J.C.D. Conducting experiments: N.A., A.S.N., A.C., O.S.W., T.D.P., and R.A.R.‐M. Analyzing data: N.A., R.A.R.‐M., J.V., and J.C.D. Manuscript drafting and review: N.A., A.S.N., A.C., O.S.W., T.D.P., R.A.R.‐M., J.V., C.P.N., and J.C.D.

## Funding

This work was supported by NIH (R00AG057825, R01AG080731, and K02AG088474) to J.C.D. and NIH (R00AG070104) to C.P.N. Phosphoproteomic work was supported by the Protein Phenotypes of Aging Core at the UW Nathan Shock Center (NIH/NIA P30AG013280) through a Pilot award to J.C.D. and by NIH/NIGMS R35GM152061 to J.V. A.C. was supported by a Virginia Tech IGEP: Translational Obesity and Healthspan Fellowship.

## Conflicts of Interest

The authors declare no conflicts of interest.

## Supporting information


**Figure S1:** Principle component analysis of p‐proteomics data illustrating that groups separate as a function of both age and exercise.
**Figure S2:** (A) Representative immunoblots of p62, total Drp1, and Drp1 phosphorylated at Ser616 in whole lysates from GA muscle of young (3 month) and old (24 month) male mice that remained sedentary (Sed) or were sampled 0 h or 6 h after a single work‐normalized bout of treadmill exercise, with Ponceau S staining as loading control. (B) p62 abundance normalized to Ponceau S (main effect of exercise, *p* < 0.001; main effect of age, *p* < 0.001). (C) Drp1 Ser616 phosphorylation normalized to total Drp1 (main effect of exercise, *p* < 0.05). (D) Representative immunoblot of Drp1 in enriched mitochondrial fractions from GA muscle, with Ponceau S staining as loading control. (E) Mitochondrial Drp1 abundance normalized to Ponceau S (main effect of age, *p* < 0.05).
**Figure S3:** GO pathway analysis of significantly downregulated phosphorylation events unique to 24‐month‐old mice with acute exercise.
**Figure S4:** (A) GA weight with age. (B) GA mass correlated to mitoAMPK⍺1 or (C) mitoAMPK⍺2 expression normalized to VDAC. (D) Immunoblots of mitoAMPK isoforms at 6, 12, 18, and 24 months of age in whole tissue lysates of GA muscles. (E, F) Quantification of mitoAMPK⍺1 and mitoAMPK⍺2 at 6, 12, 18, and 24 months of age in whole tissue lysates of GA muscles.
**Figure S5:** (A) Purification of recombinant full‐length TRIM72 protein was shown on a 10% Tricine‐PAGE Gel. Lane order was ladder (L), uninduced (1), induced (2), lysate (3), pellet (4), flow through (5), pure SUMO‐TRIM72 (6). (B) Western blot of the accompanying TCA‐stained gel identifying purified SUMO‐TRIM72.Lane order was ladder (L), uninduced (1), induced (2), lysate (3), pellet (4), flow through (5), pure SUMO‐TRIM72 MW of SUMO‐TRIM72 ~65 kDa. (C) Immunoblot of enriched mitochondria fraction from TRIM72 treated muscle lysates. (D, E) Quantification of mitoAMPK⍺1 and mitoAMPK⍺2. ATP5A (Complex V) and MTCO1 (Complex IV) were assessed as mitochondrial protein controls. Data presented as mean ± SD. Results of one‐way anova (D, E) are reported in each panel where applicable.
**Figure S6:** (A) Body mass of acute exercised young cohort (3 month, *n* = 5) and old (24 month, *n* = 17) male mice. (B) Work normalization across the groups. Exercised young mice (*n* = 5) served as the reference group and speed was adjusted for old mice based on the ratio of the average young body weight to each old mouse's body weight.

## Data Availability

The data within this article are available in the article and in its Supporting Information.
