# Supplementary material for: Impaired Exercise‐Induced Mitophagy in Aged Muscle Coincides With Altered mitoAMPKα in Male Mice
Source: Aging Cell. 2026 Nov 9;25(11):e70827. doi: 10.1111/acel.70827 (PMC13748706; doi:10.1111/acel.70827)
Supplement: Supplementary file 1 [file ACEL-25-e70827-s001.zip › Supp.docx]

**Supplemental Figures**

**Supplemental Figure S1.** Principle component analysis of p-proteomics data illustrating that groups separate as a function of both age and exercise.

**Supplemental Figure S2.** (A) Representative immunoblots of p62, total Drp1, and Drp1 phosphorylated at Ser616 in whole lysates from GA muscle of young (3 mo) and old (24 mo) male mice that remained sedentary (Sed) or were sampled 0 h or 6 h after a single work-normalized bout of treadmill exercise, with Ponceau S staining as loading control. (B) p62 abundance normalized to Ponceau S (main effect of exercise, p < 0.001; main effect of age, p < 0.001). (C) Drp1 Ser616 phosphorylation normalized to total Drp1 (main effect of exercise, p < 0.05). (D) Representative immunoblot of Drp1 in enriched mitochondrial fractions from GA muscle, with Ponceau S staining as loading control. (E) Mitochondrial Drp1 abundance normalized to Ponceau S (main effect of age, p < 0.05). Data are presented as mean ± SD. Main or interaction effects from Two-way ANOVA are reported in each panel where applicable.

**Supplemental Figure S3.** GO pathway analysis of significantly downregulated phosphorylation events unique to 24-month-old mice with acute exercise.

**Supplemental Figure S4.** A) GA weight with age. B) GA mass correlated to mitoAMPK⍺1 or C) mitoAMPK⍺2 expression normalized to VDAC. D) Immunoblots of mitoAMPK isoforms at 6, 12, 18, and 24 months of age in in whole tissue lysates of GA muscles. E-F) Quantification of mitoAMPK⍺1 and mitoAMPK⍺2 at 6, 12, 18, and 24 months of age in whole tissue lysates of GA muscles. n = 6 per age.

**Supplemental Figure S5.** A) Purification of recombinant full-length TRIM72 protein was shown on a 10% Tricine-PAGE Gel. Lane order was ladder (L), uninduced (1), induced (2), lysate (3), pellet (4), flow through (5), pure SUMO-TRIM72 (6). B) Western blot of the accompanying TCA-stained gel identifying purified SUMO-TRIM72.Lane order was ladder (L), uninduced (1), induced (2), lysate (3), pellet (4), flow through (5), pure SUMO-TRIM72 MW of SUMO-TRIM72 ~65kDa C) Immunoblot of enriched mitochondria fraction from TRIM72 treated muscle lysates. D, E) Quantification of mitoAMPK⍺1 and mitoAMPK⍺2. ATP5A (Complex V) and MTCO1 (Complex IV) were assessed as mitochondrial protein controls. Data presented as mean ± SD. Results of one-way anova (D, E) are reported in each panel where applicable. ** = p < 0.01; *** = p < 0.001

**Supplemental Figure S6.** (A) Body mass of acute exercised young cohort (3 month, n=5) and old (24 month, n=17) male mice. (B) Work normalization across the groups. Exercised young mice (n = 5) served as the reference group and speed was adjusted for old mice based on the ratio of the average young body weight to each old mouse’s body weight. Data presented as mean ± SD. Groups were compared by unpaired Student's t-test. *** = p < 0.001.
