# Supplementary material for: Impaired Exercise‐Induced Mitophagy in Aged Muscle Coincides With Altered mitoAMPKα in Male Mice
Source: Aging Cell. 2026 Nov 9;25(11):e70827. doi: 10.1111/acel.70827 (PMC13748706; doi:10.1111/acel.70827)
Supplement: Supplementary file 1 [file ACEL-25-e70827-s001.zip › Supp.pdf]

Supplemental Figures

Figure S1

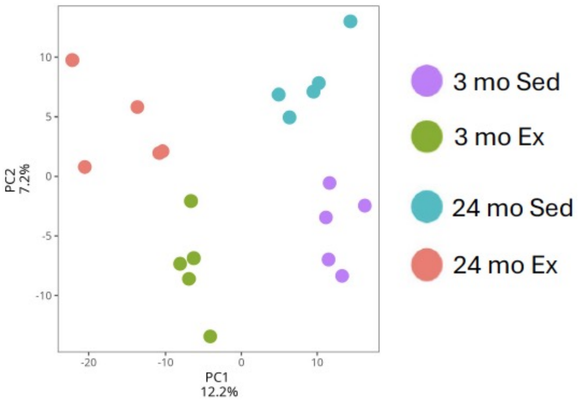

Figure S2

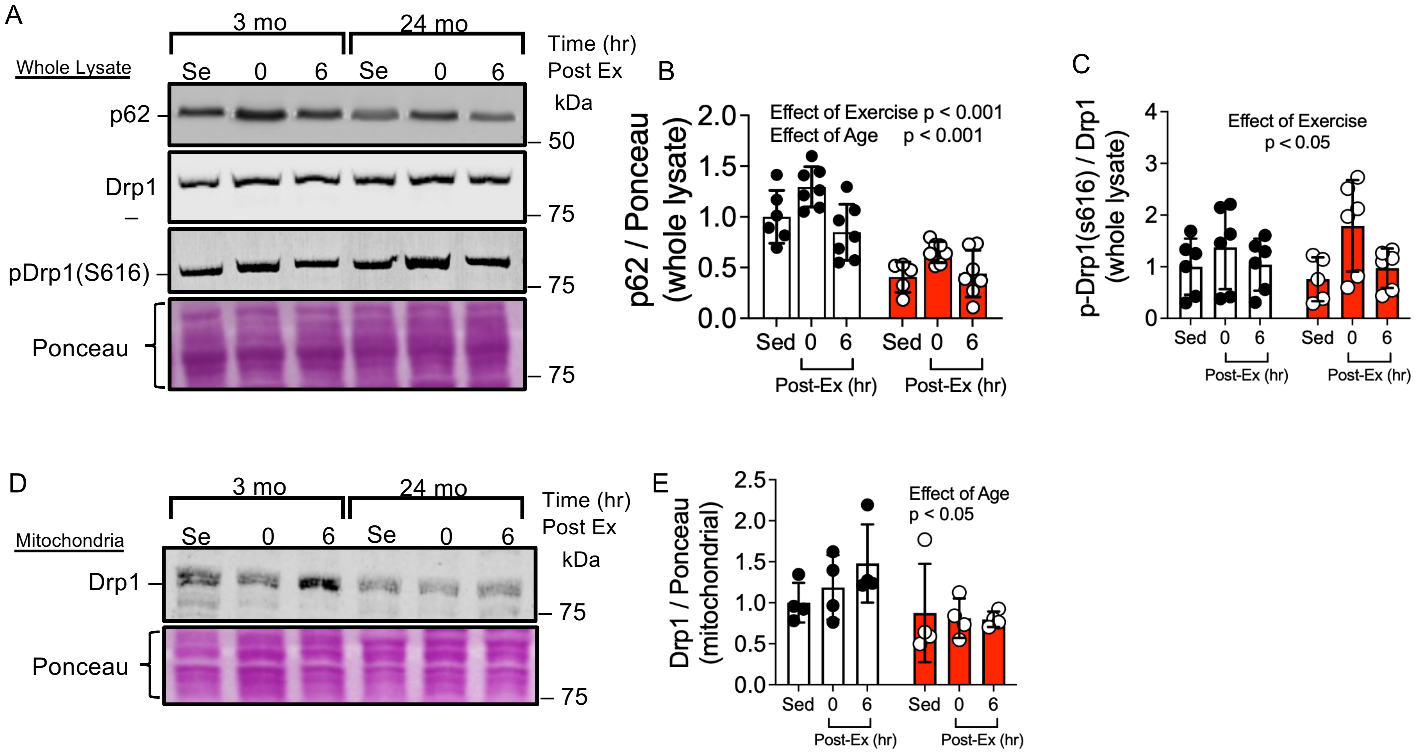

A
Whole Lysate
3 mo
24 mo
Time (hr)
Post Ex
Se 0 6 Se 0 6
kDa
p62
50
Drp1
75
pDrp1(S616)
75
Ponceau
75
B
Effect of Exercise p < 0.001
Effect of Age p < 0.001
p62 / Ponceau
(whole lysate)
Sed 0 6 Sed 0 6
Post-Ex (hr) Post-Ex (hr)
C
Effect of Exercise
p < 0.05
p-Drp1(s616) / Drp1
(whole lysate)
Sed 0 6 Sed 0 6
Post-Ex (hr) Post-Ex (hr)
D
Mitochondria
3 mo
24 mo
Time (hr)
Post Ex
Se 0 6 Se 0 6
kDa
Drp1
75
Ponceau
75
E
Effect of Age
p < 0.05
Drp1 / Ponceau
(mitochondrial)
Sed 0 6 Sed 0 6
Post-Ex (hr) Post-Ex (hr)

Figure S3

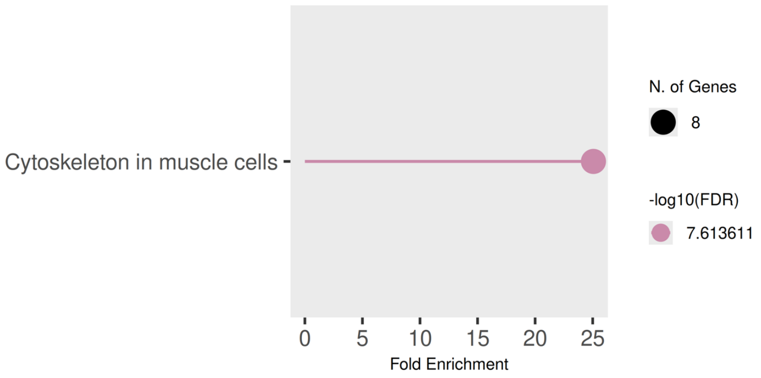

Cytoskeleton in muscle cells
0
5
10
15
20
25
Fold Enrichment
N. of Genes
8
-log10(FDR)
7.613611

Figure S4

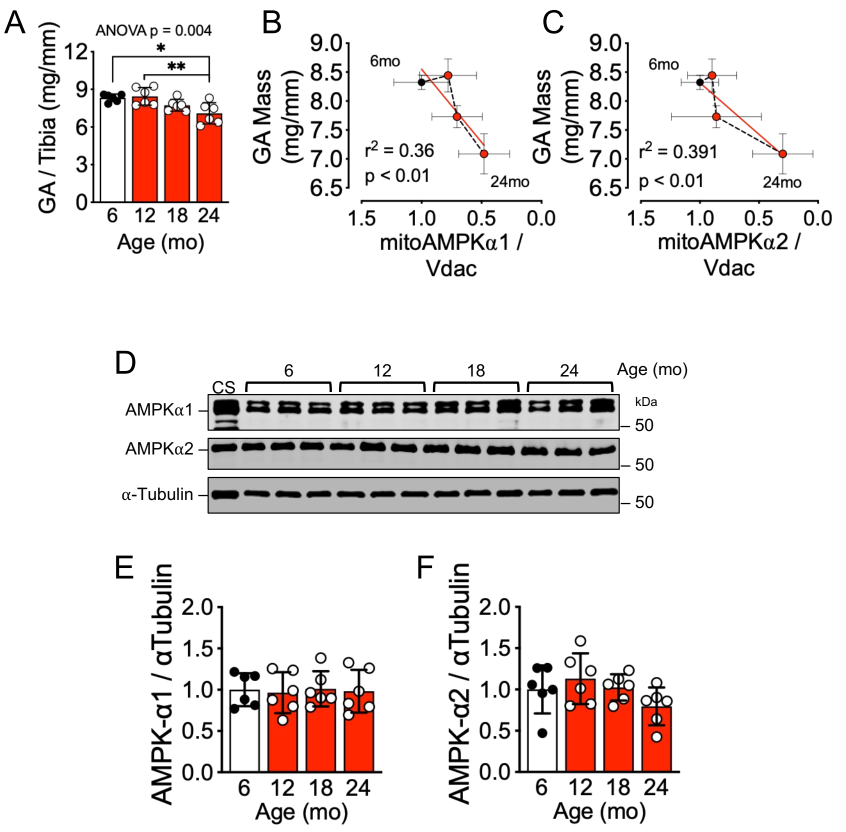

A
ANOVA p = 0.004
*
**
GA / Tibia (mg/mm)
12
9
6
3
0
6 12 18 24
Age (mo)
B
GA Mass (mg/mm)
9.0
8.5
8.0
7.5
7.0
6.5
6mo
24mo
r2 = 0.36
p < 0.01
1.5 1.0 0.5 0.0
mitoAMPKα1 / Vdac
C
GA Mass (mg/mm)
6mo
24mo
r2 = 0.391
p < 0.01
1.5 1.0 0.5 0.0
mitoAMPKα2 / Vdac
D
CS
6
12
18
24
Age (mo)
kDa
AMPKα1
AMPKα2
α-Tubulin
50
E
AMPK-α1 / αTubulin
2.0
1.5
1.0
0.5
0.0
6 12 18 24
Age (mo)
F
AMPK-α2 / αTubulin
6 12 18 24
Age (mo)

Figure S5

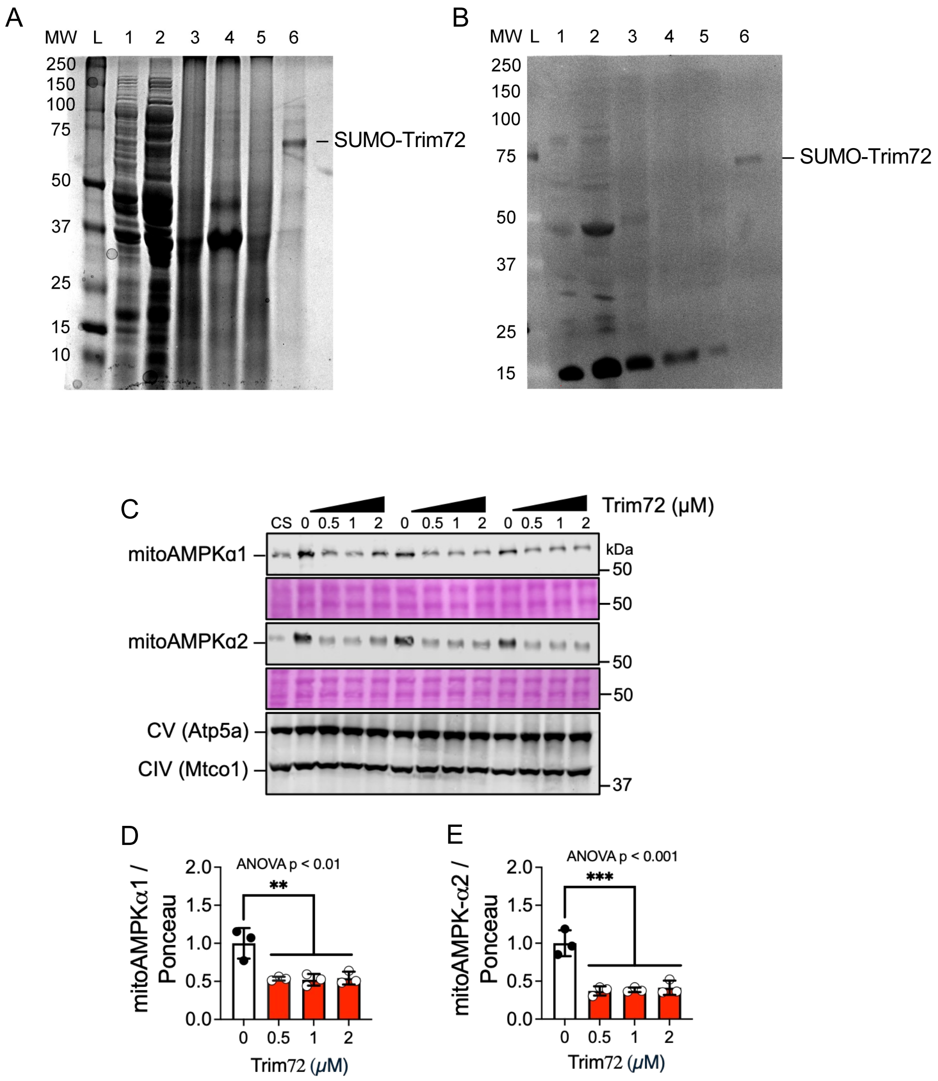

A
MW L 1 2 3 4 5 6
250
150
100
75
50
37
25
15
10
SUMO-Trim72
B
MW L 1 2 3 4 5 6
250
150
100
75
50
37
25
15
SUMO-Trim72
C
Trim72 (μM)
CS 0 0.5 1 2 0 0.5 1 2 0 0.5 1 2
mitoAMPKα1
kDa
50
50
mitoAMPKα2
50
50
CV (Atp5a)
CIV (Mtco1)
37
D
ANOVA p < 0.01
**
mitoAMPKα1 / Ponceau
2.0
1.5
1.0
0.5
0.0
0 0.5 1 2
Trim72 (μM)
E
ANOVA p < 0.001
***
mitoAMPK-α2 / Ponceau
2.0
1.5
1.0
0.5
0.0
0 0.5 1 2
Trim72 (μM)

Figure S6

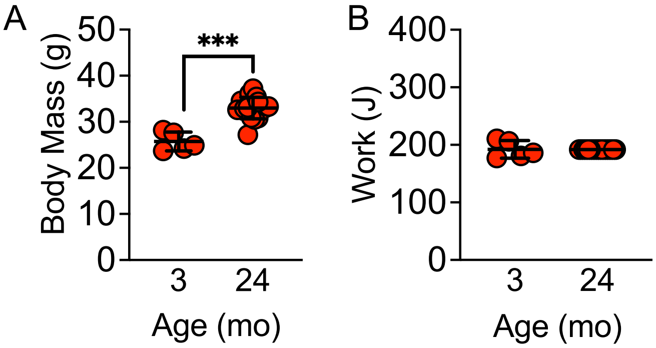
A
Body Mass (g)
50
40
30
20
10
0
***
3
24
Age (mo)
B
Work (J)
400
300
200
100
0
3
24
Age (mo)
